# The role of self-efficacy in women’s autonomy for health and nutrition decision-making in rural Bangladesh

**DOI:** 10.1186/s12889-024-17663-2

**Published:** 2024-02-01

**Authors:** Allison P. Salinger, Ellen Vermes, Jillian L. Waid, Amanda S. Wendt, Sarah J. N. Dupuis, Md Abul Kalam, Abdul Kader, Sheela S. Sinharoy

**Affiliations:** 1 Hubert Department of Global Health, Rollins School of Public Health, Emory University, Atlanta, GA 30322 USA; 2 Department of Epidemiology, Rollins School of Public Health, Emory University, Atlanta, GA USA; 3 Research Department 2, Potsdam Institute of Climate Impact Research, Member of the Leibniz Association, Potsdam, Germany; 4 Heidelberg Institute of Global Health, Heidelberg University, Heidelberg, Germany; 5 Bangladesh Country Office, Helen Keller International, Dhaka, Bangladesh; 6 Global Health and Development Program, Laney Graduate School, Emory University, Atlanta, GA USA

**Keywords:** Women’s empowerment, Agency, Preference, Measurement, Agriculture

## Abstract

**Background:**

Agency – including the sub-domains of intrinsic agency, instrumental agency, and collective agency – is a critical component of the women’s empowerment process. Self-efficacy (a component of intrinsic agency) may operate as a motivational influence for women to make choices according to their own preferences or goals, such that higher self-efficacy would be associated with more autonomous decision-making (a key component of instrumental agency).

**Methods:**

We examine these relationships using mixed methods. We developed a series of decision-making autonomy indices, which captured alignment between the woman’s reported and preferred roles in health and nutrition decisions. Using ordinal logistic regression, we assessed the relationship between generalized self-efficacy and decision-making autonomy.

**Results:**

There was a consistently positive association across all categories of decision-making, controlling for a number of individual and household-level covariates. In a sub-sample of joint decision-makers (i.e., women who reported making decisions with at least one other household member), we compared the association between generalized self-efficacy (i.e., one’s overall belief in their ability to succeed) and decision-making autonomy to that of domain-specific self-efficacy (i.e., one’s belief in their ability to achieve a specific goal) and decision-making autonomy. Across all decision-making categories, domain-specific self-efficacy was more strongly associated with decision-making autonomy than generalized self-efficacy. In-depth interviews provided additional context for interpretation of the regression analyses.

**Conclusions:**

The results indicate the importance of the role of self-efficacy in the women’s empowerment process, even in the traditionally female-controlled areas of health and nutrition decision-making. The development of the decision-making autonomy index is an important contribution to the literature in that it directly recognizes and captures the role of women’s preferences regarding participation in decision-making.

**Supplementary Information:**

The online version contains supplementary material available at 10.1186/s12889-024-17663-2.

## Background

Women’s empowerment and involvement in household decision-making have been shown to have important consequences for children’s nutrition: studies in Bangladesh have shown that children of mothers with less involvement in decision-making have an increased risk of low birthweight and increased likelihood of stunting, being underweight, and wasting than children of mothers who are more involved in decision-making [1–3]. However, there is debate among researchers concerning how best to measure women’s decision-making. One systematic review determined that studies attempting to draw causal linkages between women’s empowerment and child nutrition have been largely inconclusive due to issues with study design, including the need for more precise and transparent measurement of women’s empowerment and its domains [4]. Others have concluded that involvement in different types or domains of decision-making (e.g., healthcare decisions, family planning decisions, and purchasing decisions) had differential impact on child nutrition outcomes [5]. While general decision-making measures are common, few studies have focused on specific areas of decision-making [5]. We have, therefore, proposed a decision-making index based on the theoretical frameworks laid out below which accounts for many of the issues discussed here. We examine the utility of this measure and assess whether self-efficacy may be associated with women’s autonomy in health and nutrition decision-making.

**Fig. 1:**
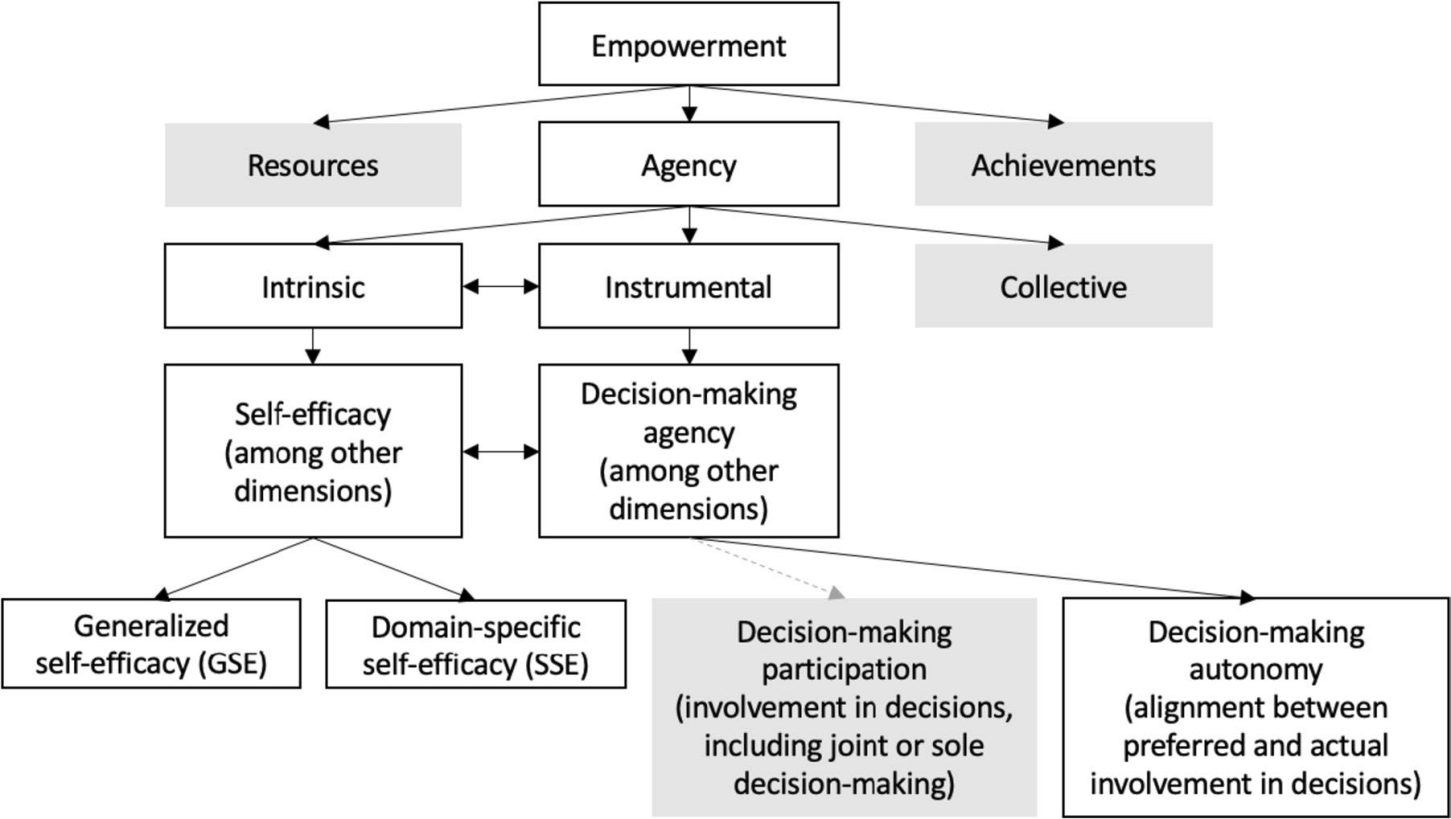
Relationships between concepts of empowerment tested in this study. Note: Grey boxes represent concepts that are outside the scope of this study. The grey, dotted line from decision-making agency to decision-making participation represents our argument that these measures do not sufficiently capture decision-making agency

Empowerment is defined as “the expansion in people’s ability to make strategic life choices in a context where this ability was previously denied to them” [6]. The process of empowerment involves access and ability to claim *resources* (i.e., material, human, and social capital); expansion of *agency* (via involvement in decision-making processes or negotiation); and ultimately, improved outcomes or *achievements* [6]. While measures of resources provide an indication of *potential choice*, elements of agency must be introduced in order to capture *actualized choice*. Measurement of achievement alone is also problematic because few achievements are universally valued across various cultural contexts [6, 7]. Therefore, researchers of women’s empowerment have historically emphasized the measurement of agency, which can be further divided into intrinsic agency (i.e., process by which women develop critical consciousness of their own goals or aspirations and capabilities) and instrumental agency (i.e., strategic action women take toward such goals) [8, 9] (See Fig. 1). Intrinsic agency is thus aligned with Rowlands’ “power within” and instrumental agency with “power to” [6, 10, 11]. Of these sub-domains, intrinsic agency has been historically understudied as have the relationships between intrinsic and instrumental agency [12].

Self-efficacy is defined as an individual’s belief in their ability to execute action toward a certain task or goal [13]. Individuals develop efficacy expectations (i.e., beliefs about whether they can successfully carry out a behavior or achieve a task/goal) and outcome expectations (i.e., beliefs about the outcome of the given behavior, task, or goal) based on personal mastery experiences, vicarious experiences, verbal persuasion, and/or emotional arousal [14]. Mastery experiences, or personal experiences with success in a given area, have been shown to have the strongest impact, of the four aforementioned sources of information, on expectations and perceived self-efficacy [14, 15]. Self-efficacy is a motivational construct; proponents of its theoretical basis would argue that belief in one’s own capabilities (or intrinsic agency) would motivate action to achieve one’s goals and fortify staying power or resolve in the face of obstacles [13]. There is precedent in the literature for assessment of self-efficacy as a predictor or component of women’s empowerment [12, 16, 17].

Researchers of both agency and self-efficacy debate the merits of and relationship between generalized and domain-specific measures [12, 18–20]. Generalized self-efficacy (GSE), which theoretically operates as a trait, is said to be the product of cumulative successes and failures across the lifespan and across several task domains, and is shown to be more resistant to external influences [19, 21]. By contrast, domain-specific self-efficacy (SSE) refers to one’s belief in their ability to achieve a specific task or find success in a defined domain or area of life [18]. Some have argued that GSE has a top-down effect on SSE meaning GSE operates as an important predictor of SSE across various task domains [18, 19]. Others have argued that cumulative SSE across task domains has a bottom-up effect on GSE or that there is a reciprocal relationship between these two forms of self-efficacy [20, 22]. For this reason, our study incorporates and compares measures of both generalized and domain-specific self-efficacy.

Attempts to assess women’s empowerment have typically focused not only on agency, but specifically on *decision-making agency*, as an observable behavior indicative of women’s instrumental agency and/or empowerment [5, 12, 23, 24]. Measures of decision-making agency usually ask about either participation in various household decisions captured as a binary value (i.e., did or did not participate) or, more recently, the degree to which women participate in household-level decision-making processes [25–27]. These types of questions may be more precisely classified as measures of women’s *decision-making participation*, which has been linked to improved outcomes for children’s health and nutrition and is generally accepted as an indicator of women’s empowerment [28]. There has also been a focus on sole decision-making (i.e., woman makes decision alone) versus joint decision-making (i.e., woman makes decision with spouse or with at least one other household member). Some argue that only sole decision-making constitutes agency in household decisions [29] while others posit that sole decision-making can actually create an additional burden for women [30, 31]. At the same time, joint decision-making is often unequal and may involve no discussion or discussions in which women voice opinions but men have the final say [25, 30].

More participation in decision-making is not necessarily preferred in all cases. Women’s decision-making participation is only pertinent to women’s agency (and thus, women’s empowerment) if women *prefer* increased participation. The concept of autonomy refers to a woman’s *ability* to influence decisions that affect her and her family if she so chooses or rather if she has an interest or finds value in participating in such decisions [32, 33]. Indeed, self-determination theory argues that those who are autonomously motivated (rather than motivated by avoiding punishment, guilt, or shame) exhibit behaviors that are aligned with their own values and interests [34]. Measuring participation in decision-making then is insufficient to assess women’s agency; we must also capture whether women valued being or wanted to be involved. To this end, empowerment researchers have called for measures of decision-making agency that account for women’s preferences [10, 25, 27, 35]. One study examined how the observed effect on key program outcomes changed depending on the indicator of women’s decision-making used in analysis (i.e., sole decision-making, sole or joint decision-making, sole decision-making after disagreement) [35]. The authors found a low association between women’s “ideal decision-making arrangement” and any of the decision-making indicators [35]. Indicators that are pre-determined by researchers to be reflections of agency or empowerment can be entirely misaligned with women’s preferred roles in decision-making [35]. Accordingly, we propose an examination of decision-making autonomy (i.e., alignment between preferred involvement and experienced involvement) rather than decision-making participation (i.e., degree of involvement in decision-making processes) using an index similar to that presented by Peterman et al. to assess women’s instrumental agency (See Fig. 1).

The aim of this study was to examine associations between women’s intrinsic and instrumental agency, as measured through perceived self-efficacy and decision-making autonomy, respectively. We hypothesized that self-efficacy (a proxy for intrinsic agency) would be associated with instrumental agency such that women with more self-efficacy are better able to act according to their preferences in household decision-making regarding health and nutrition. The study’s objectives are to (1) introduce an index of decision-making autonomy that captures not only involvement in decision-making but that also directly recognizes and accounts for the role of women’s preferences regarding participation in decision-making, (2) assess whether GSE is associated with women’s decision-making autonomy, (3) assess whether GSE or SSE is more strongly associated with women’s decision-making autonomy, among joint decision-makers (i.e., women who reported making decisions with at least one other household member) for whom SSE data were available, and (4) interpret this relationship within the context of women’s lived experiences in rural Bangladesh using qualitative data.

## Methods

This cross-sectional study using secondary data followed a sequential, mixed-methods design in which findings from the quantitative analyses informed the direction of the qualitative analyses.

### Study design & intervention background

This study used existing data from the Food and Agricultural Approaches to Reducing Malnutrition (FAARM) trial (ClinicalTrials.gov: NCT02505711). FAARM was one of 13 projects across Africa and South Asia involved in the Gender, Agriculture, and Assets Project – Phase 2 (GAAP2). GAAP2 partners collaboratively developed the tools utilized in this study, including the pro-WEAI and accompanying modules, discussed below.

FAARM was a cluster-randomized controlled trial designed to determine the impact of a homestead food production (HFP) intervention on women’s and children’s nutritional status. The intervention was implemented by Helen Keller International from 2015 to 2018 in rural Habiganj District, Sylhet Division, Bangladesh. Further information about the setting and the design of the FAARM trial is available in the study protocol [36]. In brief, households were eligible for enrollment in the FAARM trial if there was a woman under the (self-reported) age of 30, who had access to at least 40 square meters of land and who was married to a husband who spent the night at home at least once in the previous year. After eligible women were identified, 96 settlements (villages or sections of villages) of 10-65 eligible women were formed; these settlements were then randomly assigned to intervention and control arms. Overall, approximately 2,700 women were enrolled in the trial [36].

The HFP intervention was implemented through women farmer groups; group members selected a lead farmer family who was then tasked with overseeing the group’s model farm, producing and distributing seeds and seedlings, hosting regular meetings, and facilitating training. Women’s empowerment was one pathway through which it was hypothesized that the intervention would lead to improved nutritional outcomes. Specifically, certain intervention activities were intended to transfer assets, knowledge, and skills to women; to increase women’s control over productive resources; and to create opportunities for women’s group participation and leadership [36].

### Quantitative data collection

The main pro-WEAI [17] and modified health and nutrition modules [37] were administered in April and May 2019 (4-5 months after the intervention was completed). Detailed methods for the survey are provided elsewhere [38]; information relevant to the current study is summarized below.

The survey targeted five households in each of the 96 FAARM trial settlements for a total sample of 480 households. Targeted households were randomly selected from a list of households with fewer than 10 members and only one woman enrolled in FAARM at baseline (for ease of survey administration). Four female enumerators were trained and administered the survey in Bengali to the enrolled woman in each selected household. Written informed consent by signature, or thumbprint and the signature of an independent witness (i.e., a subject that is not part of the study team) if the subject was illiterate, was obtained from all FAARM participants at the beginning of the trial in 2015.

The pro-WEAI survey tool was designed to identify and evaluate key empowerment outcomes of agricultural development interventions [17]. The tool consists of 12 indicators representing three domains of agency: intrinsic agency, instrumental agency, and collective agency. Self-efficacy is classed as an indicator of intrinsic agency while most decision-making indicators fall under instrumental agency. The main pro-WEAI includes decisions related only to the productive sphere (i.e., agricultural and income-related decisions) while the supplemental health and nutrition module - designed to measure aspects of instrumental agency more directly related to health and nutrition - includes decisions related to the domestic sphere [37]. Our study focuses on 15 decision-making topics/activities (i.e., diet, nutrition, health, and healthcare seeking behaviors related to the woman respondent herself or to her children), which were captured by the health and nutrition module (See Supplementary Table A). The pro-WEAI also includes the 8-item New General Self-Efficacy scale (NGSE), which was used to generate a GSE score for each woman, as described below.

Some of the demographic data used as control variables in the final models was collected during the FAARM baseline survey, which was administered to all enrolled women from March to May 2015. Additional information on the FAARM baseline survey is available elsewhere [39].

### Quantitative data analysis

#### Measures

The NGSE scale includes statements about generalized self-efficacy (e.g., “*I will be able to achieve most of the goals I have set for myself*”) to which the respondent indicates level of agreement on a 5-point, Likert-type response scale that ranges from strongly disagree to strongly agree. The GSE scores for each respondent were summed for a total possible score of 40, which was used in our models as a continuous measure [18].

To assess participation in decision-making, women were asked, *“When decisions are made about [activity], who normally takes the decision?”* Women who listed themselves as the only decision-maker for a given decision were classified as sole decision-makers for the given activity. Women who listed themselves and at least one other household member were classified as joint decision-makers for the given activity. Only joint decision-makers were then asked, “*How confident do you feel to make decisions about [activity]?*” This question, which we used as a measure of SSE, had an ordinal response scale of not at all, somewhat, and very confident. Very few women reported that they were not at all confident for any of the health and nutrition activities. Therefore, we assigned a value of 0 to not at all and somewhat confident responses and a value of 1 to very confident responses. These values were then summed across the three activities assigned to each of five activity categories (personal health and diet; personal diet during pregnancy; personal health during pregnancy; child’s diet; healthcare seeking) (See Supplementary Table A for the full list of domain-specific self-efficacy items and the indices to which they were assigned). Women were given an SSE score for each activity category ranging from 0, for describing themselves as not at all or somewhat confident across all activities, to 3 for describing themselves as very confident across all activities in a given activity category.

Decision-making autonomy was measured using a dummy variable that assessed agreement between two items: “*When decisions are made about [activity], who normally takes the decision”* and “*When decisions are made about [activity], who would you prefer made the decision?”* The decision-making autonomy variable for each activity was only calculated for the women who were involved in the relevant decision where they took a value of 1 if the woman preferred to be involved (i.e., classified as having decision-making autonomy) and a value of 0 if she did not prefer to be involved (i.e., classified as *not* having decision-making autonomy). We created decision-making autonomy indices by summing the dummy variables for the three activities within each of the five activity categories. Women were given a score ranging from 0 (non-autonomous decision-making across the category) to 3 (complete autonomous decision-making across the category) for each category. Questions regarding health and nutrition during pregnancy and about children’s diet were only asked to women with relevant experiences.

#### Regression modeling

An ordinal logistic regression modeling approach was used to account for the inherent ordering in levels of decision-making autonomy [40]. An ordinal logistic regression model (Equation 1) was estimated to assess the association between GSE and autonomous decision-making:

1 $$\mathrm{ln }\left[\frac{P(DMAUTONOMY\ge g|GSE, COV1,\dots ,COVk}{P(DMAUTONOMY<g|GSE, COV1,\dots COVk)}\right]= {\alpha }_{g}+ {\beta }_{1} X GSE+ {\gamma }_{1} X {COV}_{1}+\dots +{\gamma }_{k} X {COV}_{k}$$

Where g is equal to the number of autonomous decisions per decision-making index (1, 2, or 3) (See Supplementary Table A for the items included in each index) and where γ_1_ to γ_k_ represent control variables capturing individual and household characteristics that may have influenced self-efficacy and decision-making autonomy (i.e., intervention arm, religion, years since marriage, age at baseline, women's highest class passed, family type, and wealth decile). Wealth was calculated based on household ownership of selected assets using standard DHS techniques for the FAARM sample as a whole [39, 41]. We estimated Equation 1 for sole and joint decision-makers together, as well as for each decision-making type separately.

Another ordinal logistic regression equation (Equation 2) was then estimated to assess the association between SSE and decision-making autonomy in joint decision-makers. These analyses were conducted only for women who were joint decision-makers across all three activities in a given category. For example, if a woman reported joint decision-making in one of the questions comprising a category, but sole decision-making in the other two questions, she would not be included in the analysis for that category, due to the fact that SSE data was only available for joint decision-makers. The results for Equation 2 are also adjusted for the above-mentioned individual and household characteristics.

2 $$\mathrm{ln }\left[\frac{P(DMAUTONOMY\ge g|SSE, COV1,\dots ,COVk}{P(DMAUTONOMY<g|SSE, COV1,\dots COVk)}\right]= {\alpha }_{g}+ {\beta }_{1} X SSE+ {\gamma }_{1} X {COV}_{1}+\dots +{\gamma }_{k} X {COV}_{k}$$

No violations of the parallel regression assumption were noted for either model and analysis continued using proportional odds (cumulative logit) models [42]. All models also included robust standard errors to adjust for settlement-level clustering. All quantitative analyses were conducted using Stata v16.1 (StataCorp LP, College Station, TX, USA).

### Qualitative data collection

Detailed methods for the qualitative sampling and data collection are provided elsewhere [43]; information relevant to the current study is summarized below. In-depth interviews (IDIs) were conducted in Bengali with 12 women from 6 intervention settlements and 10 women from 5 control settlements in June and July, 2019. These settlements were randomly selected from a list of settlements where previous FAARM qualitative data collection had not taken place. Women from these settlements who had completed the quantitative pro-WEAI survey were then randomly selected and invited to participate in an IDI. Female data collectors, who were trained and experienced in qualitative research, conducted each interview in a private space either inside or directly adjacent to the interviewee’s home. The IDI question guide was designed to capture the influence of the HFP intervention on women’s individual and household experiences and changes in empowerment, including self-efficacy. The guides were piloted in an intervention settlement prior to roll-out. These guides were not explicitly designed to answer the research question that our study poses, but provide useful insights to contextualize the quantitative findings.

### Qualitative data analysis

The IDI audio recordings were transcribed verbatim in Bengali and the transcripts were then translated into English. A codebook was developed deductively using key concepts of interest from the quantitative analysis. The codebook consisted of several codes that were each assigned to one of four domains: self-efficacy, decision-making, agency (other than decision-making), and sociocultural context. The transcripts used in this analysis were previously coded as part of a grounded theory analysis that sought to describe the pathway to women’s empowerment through the HFP intervention [43]. Codes used in the previous analysis, which were developed inductively, were leveraged in our analysis where relevant. All coding was conducted in MAXQDA2020 (VERBI Software, 2019). Coded text segments were then reviewed to identify patterns in the ways in which the 22 women in the sample experienced, perceived, and spoke of the four key domains. This thematic analysis yielded in-depth descriptions of each of the four key domains; the themes most relevant for answering the research questions posed above are discussed in the qualitative results section below [44].

## Results

### Quantitative results

#### Population characteristics and decision-making outcomes

Approximately one-third (37%) of women were pregnant or had given birth within the two years prior to the pro-WEAI survey, and 79% of women had children in their household who were at least 6 months of age. The mean GSE score for women in our study population was 29.6 (out of a total possible score of 40) (Table 1).

**Table 1:** Characteristics of women included in the pro-WEAI survey (*n*=457)

**Variable**	**freq (%) or Mean (SD) or Median (Range)**
Self-efficacy	29.6 (4.4)
Intervention arm	230 (50.3%)
Pregnant or given birth in past 2 years	169 (37.0%)
Child ≥ 6 months of age in household	361 (79.0%)
Religion^a^	
*Muslim*	331 (72.4%)
*Hindu*	126 (27.6%)
Parity†	3 (1-10)
Age^a^	24.9 (4.2)
Age at time of marriage^a^	17.9 (2.4)
Age gap (husband-wife)^a^	8.2 (5.5)
Education gap in class years completed (husband-wife)^a^	-1.1 (3.4)
Years since marriage^a^	7.1 (4.1)
Household size	5.6 (1.9)
Family type^a^	
*Joint*	196 (42.9%)
*Nuclear*	261 (57.1%)
Women’s education^a^	
*No formal education*	86 (18.8%)
*Part primary*	103 (22.5%)
*Full primary*	96 (21.0%)
*Part secondary*	172 (37.6%)
Husband’s education^a^	
*No formal education*	193 (42.2%)
*Part primary*	76 (16.6%)
*Full primary*	76 (16.6%)
*Part secondary*	112 (24.5%)

^a^Data are from FAARM baseline survey (March-May 2015); †Data are from retrospective FAARM endline survey (October 2019 to February 2020) and apply to the time period April-May 2019; all other data were collected as part of the pro-WEAI survey (April-May 2019)

Of women involved in decision-making, autonomous decision-making was most prevalent in the personal health and diet category, where 63% of women reported preference alignment for deciding to rest when ill, 61% for deciding on foods to prepare, and 81% for deciding on foods to eat. Autonomous decision-making was least prevalent in healthcare seeking where 16% of women reported preference alignment for deciding on going to the doctor when ill, 26% for deciding on taking a sick child to the doctor, and 36% for deciding on taking a child for well-visit check-ups. A similar trend was observed for SSE. The personal health and diet category had the highest frequency of very confident responses and the healthcare seeking category had the lowest frequency of very confident responses (Table 2).

**Table 2:** Decision-making outcomes among women included in the pro-WEAI survey (*n*=457), by activity

**Decision-making category**	**n**	**Decision-making type** freq (% out of n)		**Autonomous decision-making**^a^ (% of sole and joint decision-makers)	**Decision-making confidence (SSE)** freq. (% of joint decision-makers)	
		Sole	Joint		Not at all orsomewhat confident	Very confident
**Personal health and diet**						
Rest when ill	457	283 (61.9)	169 (37.0)	286 (63.3)	70 (41.4)	99 (58.6)
Foods to prepare	457	319 (69.8)	134 (29.3)	274 (60.5)	47 (35.0)	87 (65.0)
Foods to eat	457	386 (84.5)	68 (14.9)	366 (80.6)	27 (39.7)	41 (60.3)
**Personal diet during pregnancy**						
Eat eggs during pregnancy^c^	169	80 (47.3)	84 (49.7)	87 (53.0)	37 (44.0)	47 (56.0)
Consume milk during pregnancy^c^	169	63 (37.3)	104 (61.5)	81 (48.5)	58 (55.8)	46 (44.2)
Eat meat during pregnancy^c^	169	50 (29.6)	118 (69.8)	67 (39.9)	79 (67.0)	39 (33.0)
**Personal health during pregnancy**						
Work during pregnancy^c^	169	60 (35.5)	106 (62.7)	81 (48.8)	52 (49.0)	54 (51.0)
Rest during pregnancy^c^	169	79 (46.7)	85 (50.3)	94 (57.3)	38 (44.7)	47 (55.3)
Consult a doctor during pregnancy^c^	169	7 (4.1)	157 (92.9)	29 (17.7)	108 (68.8)	49 (31.2)
**Child’s diet**						
Feed child eggs^d^	361	168 (46.5)	182 (50.4)	207 (59.3)	84 (46.4)^b^	97 (53.6)^b^
Feed child milk^d^	361	137 (37.9)	221 (61.2)	205 (57.3)	108 (48.9)	113 (51.1)
Feed child meat^d^	361	102 (28.2)	252 (69.8)	143 (40.5)	162 (64.5)^b^	89 (35.5)^b^
**Healthcare seeking**						
Go to the doctor when ill	457	15 (3.3)	437 (95.6)	72 (15.9)	300 (68.7)	137 (31.3)
Take sick child to doctor^e^	434	16 (3.7)	412 (94.9)	110 (25.7)	223 (54.1)	189 (45.9)
Take child for well visits^e^	434	57 (13.1)	373 (85.9)	153 (35.6)	200 (53.6)	173 (46.4)

^a^Binary dummy variable calculated for the women who were involved in the relevant decision as either sole or joint decision-makers that took a value of 1 if the woman preferred to be involved (i.e., classified as having decision-making autonomy)

^b^1 missing value, may not sum to total

^c^asked to women who were pregnant or had given birth within the past 2 years

^d^asked to women who had children at least 6 months of age

^e^asked to women with children

#### Associations between GSE and decision-making autonomy

Women with greater generalized self-efficacy were more likely to have higher decision-making autonomy in personal health and diet (OR: 1.07; 95% CI: 1.02, 1.12) (Table 3). Similar trends were apparent in relation to child’s diet and healthcare seeking: for a one-unit increase in GSE score, the odds of having higher decision-making autonomy increased by 14% (95% CI: 1.08, 1.21) and 15% (95% CI: 1.10, 1.21), respectively. For personal diet during pregnancy and personal health during pregnancy, there was no evidence of an association.

**Table 3:** Association between generalized self-efficacy (GSE) and autonomous decision-making for sole and joint decision-makers

	**Decision-making autonomy index**				
	**Personal health and diet (*N*= 445)**	**Personal diet during pregnancy (*N*= 161)**	**Personal health during pregnancy (*N*= 159)**	**Child’s diet (*N*= 347)**	**Healthcare seeking (*N*= 424)**
**General self-efficacy**					
OR (95 % CI)	1.07 (1.02-1.12)	1.05 (0.96-1.14)	1.01 (0.94-1.09)	1.14 (1.08-1.21)	1.15 (1.10-1.21)
*p*-value	0.004	0.33	0.70	<0.001	<0.001

Results are from ordinal logistic regression models controlling for intervention arm, religion, years since marriage, age at baseline, women's highest class passed, family type, wealth decile, and adjusted for settlement-level clustering

#### Influence of GSE vs SSE on autonomy among joint decision-makers

We also examined the relationship between GSE and autonomous decision-making (Table 4) and between SSE and autonomous decision-making (Table 5). These analyses were conducted for women who were joint decision-makers across all three activities in a given category. Among joint decision-makers, domain-specific self-efficacy was more strongly associated with decision-making autonomy than generalized self-efficacy.

**Table 4:** Association between generalized self-efficacy (GSE) and autonomous decision-making for joint decision-makers

**Variable**	**Decision-making autonomy index**				
	**Personal health and diet (*N*= 44)**	**Personal diet during pregnancy (*N*= 73)**	**Personal health during pregnancy (*N*= 79)**	**Child’s diet (*N*= 159)**	**Healthcare seeking (*N*= 365)**
**General self-efficacy**					
OR (95 % CI)	1.01 (0.86-1.17)	1.03 (0.92-1.15)	1.03 (0.89-1.19)	1.13 (1.06-1.22)	1.15 (1.09-1.22)
*p*-value	0.94	0.66	0.70	<0.001	<0.001

Results are from ordinal logistic regression models controlling for intervention arm, religion, years since marriage, age at baseline, women's highest class passed, family type, wealth decile, and adjusted for settlement-level clustering

**Table 5:** Association between domain-specific self-efficacy (SSE) and autonomous decision-making in joint decision-makers

**SSE Category** (Referent category: very confident in 0 of 3 activities)		**Decision-making autonomy index**				
		**Personal health and diet (*N*= 44)**	**Personal diet during pregnancy (*N*= 73)**	**Personal health during pregnancy (*N*= 79)**	**Child’s diet (*N*= 159)**	**Healthcare seeking (*N*= 365)**
**Domain-specific self-efficacy for some activities** (very confident in 1-2 of 3 activities)	OR (95% CI)	1.06 (0.13-8.55)	0.47 (0.13-1.61)	1.37 (0.29-6.42)	2.05 (0.81-5.22)	2.69 (1.54-4.72)
	*p*-value	0.95	0.23	0.69	0.13	0.001
**Domain-specific self-efficacy for all activities** (very confident in 3 of 3 activities)	OR (95% CI)	6.46 (1.27-32.71)	4.23 (1.24-14.42)	7.54 (1.47-38.54)	8.87 (3.84-20.51)	11.62 (6.26-21.56)
	*p*-value	0.02	0.02	0.02	<0.001	<0.001

Results are from ordinal logistic regression models controlling for intervention arm, religion, years since marriage, age at baseline, women's highest class passed, family type, wealth decile, and adjusted for settlement-level clustering

### Qualitative results

#### Perceptions of self-efficacy in decision-making

In the qualitative interviews, women who were confident in their ability to participate in household decision-making often referred to mastery experiences (i.e., they were able to point to instances in which they succeeded in persuading their husbands). These women tended to have positive efficacy expectations about their ability to make effective arguments and/or positive outcome expectations about their husband’s reaction to their participation in the decision-making process.

Some women also discussed positive outcome expectations related to the specific choices they made. They were confident that their choices would lead to “fruitful” outcomes for the family. In these cases, which were usually related to decisions such as what to plant, cook, or what livestock to rear, women explained that their decisions were guided by instructions or a “formula” set out by the intervention. When women had personal mastery experiences in gardening or livestock rearing after following the “formula”, their self-efficacy to make fruitful decisions was reinforced. In some cases, this also led to increased decision-making autonomy as husbands witnessed positive household changes as a result of their wives’ decisions.

“*Earlier when I took any decision, I needed to ask my husband first. And now he understands that if I take these decisions by myself then it will be better, better for the family. So, he does not say anything now if I take decisions by myself. I do it in a good way. [...] Now he sees the income and expenditure that is happening in our family. Decisions that I take, he sees that they become fruitful for our family.*”

(Woman from Settlement A; intervention; nuclear family)

#### Socio-cultural influences on self-efficacy and decision-making

Confidence and participation in decision-making processes, however, did not necessarily translate to improved agency for all women or all decision categories. For example, most women decided what to cook for their families on their own. While women appear to have the freedom to cook what they choose, cooking was generally perceived to be the woman’s responsibility. As such, their autonomy in decision-making within this sphere of home life is not necessarily indicative of the women having agency or control over significant decisions, but rather fulfilling the role that has been assigned to them.

“*He [my husband] said that’s my job to decide what they [the children] will eat. What they will like to eat, it’s your job to take care. He will bring what they need*.”

(Woman from Settlement B; intervention; joint family)

Interviewees were asked what happens when there are disagreements or differences of opinion in the decision-making process. Many women articulated a tension between favoring logic (i.e., choosing the opinion they believed would have the best outcome for the family) and favoring alignment with social rules that dictate who has more power in the household. The social rules outlined by the interviewees included deference to men and to elders.

“Participant (P): There is some difference between a man’s word and a woman’s word. About important topics, if any elder man is saying anything, then I am younger than him. If I give any decision instead of hearing it, I will have to keep his word because I am younger than him. If [the] work’s output will be good, then there is nothing elder and younger. [...] Then he says she can be younger, but she didn’t say anything wrong. Then we can do it.

Interviewer (I): At the time of taking a decision whose decision always gets importance?

P: Most of the time, what he will say [gets importance].”

(Woman from Settlement B; intervention; joint family)

Women in joint households had even more complex household power structures. Women in joint families (i.e., living with at least one in-law) explained that they needed approval from or consultation with several family members, including mothers- and fathers-in-law and, in some cases, brothers- and sisters-in-law, before they could take action on a given decision. The social structure of joint households appeared to introduce a formality to the decision-making process and, in many cases, limited the woman’s autonomy.

“It is [more] beneficial in [a] single family than in an extended family. In an extended family, everyone takes joint decisions and eats together, I don’t want that. If the mother-in-law will agree then the father-in-law will disagree. Also, if someone agrees then the brothers-in-law will disagree. But in a single family nothing like this happens. Both of us can take a decision together. What we will think [will be] good for us, we can take that decision. We take our decision. For this reason, a single family is good.”

(Woman from Settlement A; intervention; nuclear family)

The household composition and sociocultural context in which women are deciding whether and how to participate in decision-making thus complicates the relationship between perceived self-efficacy, decision-making autonomy, and empowerment.

#### The role of adaptive preference

Some women chose to remove themselves from or limit their participation in the decision-making process because they learned that their participation created conflict or negative consequences for them within the family (i.e., they had negative outcome expectations surrounding their participation in decision-making). In other cases, the woman did voice her opinion, but conceded if her husband expressed a contrasting opinion. Other women reported lacking any opinion or preference altogether.

“If I need something and if he scolds me then I just sit down and shut my mouth. [...] When I need something, he give[s it to] me. Then I also don’t have any say. If he gives me after 2 days, I also tolerate this. Then I don’t say anything, and everything has been solved.”

(Woman from Settlement B; intervention; joint family)

“I: If there is a difference of opinion in case of spending money, what do you do?

P: I don’t have any opinion regarding the financial matters, my husband takes care of it.”

(Woman from Settlement C; intervention; nuclear family)

These examples demonstrate instances of adaptive preference in which women have either aligned their preferences for participation in decision-making with the preferences they are expected or encouraged to have (i.e., deference to their husband’s opinion) or have aligned their preferences for the outcome with the preferences of their husbands. While this appears to involve some element of choice on the part of the woman, it is not necessarily representative of her agency in cases in which she does not have the capability to select an alternative choice.

“The husband in a family has more power than anyone. He runs the family on his own decisions. [...] I have to agree even if I don’t want to. There is no point in arguing with him. If the husband orders [me] to do something, I have to do that.”

(Woman from Settlement D; control; joint family)

## Discussion

We followed a cross-sectional, mixed-methods study design to examine the relationship between self-efficacy and autonomy in health and nutrition decision-making among women enrolled in the FAARM trial in Sylhet, Bangladesh. Ordinal logistic regression modelling allowed for an assessment of the relationship between generalized self-efficacy and decision-making autonomy as well as a comparison between generalized versus domain-specific self-efficacy among women who reported making joint decisions. Qualitative thematic analysis provided a critical, emic perspective for the interpretation of the results in context.

### Decision-making autonomy

A substantial body of research in the area of women’s empowerment has sought to measure agency, and specifically, decision-making agency as an aspect of empowerment [12, 17, 23, 24, 37, 45, 46]. Authors of several of these studies have identified limitations to the current approach for capturing decision-making agency, specifically the assumption that higher participation is necessarily indicative of greater agency and thus, of empowerment. Instead, several researchers have called for a stronger focus on preference [25, 27, 35]. We incorporated preference by creating a decision-making autonomy index, similar to the indicator described in Peterman et al., 2021, that directly assesses whether women were able to act on their preferences for participation in various types of decisions.

The decision-making autonomy index used in this study accounts for women’s preferences and yielded consistent, interpretable results across various models and samples sizes. One distinct advantage of the index is that it allows for the possibility that involvement in decision-making may actually be disempowering or at least not preferred. Other tools that are used to quantify decision-making agency account for preference by asking only women who were not involved at all in the given decision whether they would have valued participating [45]. While this helps researchers avoid making the assumption that lack of involvement is necessarily disempowering, it simultaneously assumes that women who were involved in decision-making valued that decision or involvement.

Our study identified a number of women who were involved in certain decisions and preferred not to be. This is an important and often overlooked form of disempowerment that comes into play particularly when dealing with everyday decisions like the health and nutrition decisions assessed here. While it might not be problematic to assume that women value inclusion in decisions around topics like contraceptive use, marriage, etc., it is problematic to assume that women necessarily value involvement in decisions around things like foods to prepare or what to feed children. Klein gives the example of a woman who might not make “minor household choices” such as “whether to buy beans or spinach” because she and her spouse have decided that she will focus on her career and her husband on “domestic matters” [47]. Thus, it is most appropriate to assess preference alignment as a measure of decision-making autonomy (particularly for these everyday decisions) rather than pre-determining what should be considered an empowered choice [35].

The qualitative findings demonstrate that, in some cases, women can have preferences for perpetuating disempowering social norms. These adaptive preferences may represent less agency and power, as is the case when women prefer to avoid negative consequences of non-normative behavior or consent to an oppressive status quo [10, 48]. Some women may have habituated disempowering preferences around certain topics as a means of leveraging their power elsewhere [6]; these are conscious and rational adaptations as opposed to unconscious and non-autonomous adaptations [49]. While we identified some evidence of adaptive preference in the qualitative analysis, it was difficult to tease out the implications of these preferences on women’s power because it was unclear from the transcripts whether the women felt that they could have made different choices if they had so preferred.

### The role of self-efficacy in the empowerment process

We observed that generalized self-efficacy was significantly associated with decision-making autonomy, after controlling for key individual and household characteristics including intervention arm, among sole and joint decision-makers together. Domain-specific self-efficacy was also significantly associated with decision-making autonomy among joint decision-makers. Women with higher GSE or SSE scores were more likely to report that they had participated the way they wanted to participate in household decisions around health and nutrition than women with lower GSE or SSE scores. Inversely, women who were less confident about their general ability to reach their goals or about their ability to make the given decision were more likely to have participated in decision-making in a way that did not align with their preferences.

While we cannot draw causal inferences from these results, self-efficacy theory provides a possible rationale for the observed association. Researchers assert that self-efficacy operates as a motivational latent construct by which individuals with strong belief in their ability to achieve a goal are more likely to be motivated to carry out the necessary courses of action toward the goal and to maintain effort in the face of obstacles [13]. Thus, women with higher GSE or SSE may be more motivated to take the necessary action to participate or abstain from participation in decision-making than women with lower GSE or SSE. Similarly, women with higher self-efficacy may have more conviction to assert themselves to ensure their preferences are met in cases in which their spouses or mothers-in-law, for example, would prefer they participate differently.

The qualitative data suggests that mastery experiences in decision-making on health and nutrition activities may have led women to have more positive outcome expectations, and thus higher SSE, surrounding their ability to make the fruitful choice for their families. This is in line with existing research on a variety of health behaviors (e.g., cigarette smoking, weight control, contraception, alcohol abuse and exercise behaviors), which suggests that mastery, or personal experience with success increases self-efficacy [14, 15]. Locus of control is a similar and related concept that refers to “the degree to which an individual believes that events are caused by one’s own behavior (internal locus of control) versus external factors (external locus of control)” [12, 50]. Certain health and nutrition decisions necessarily involve more external variables. For example, healthcare seeking decisions require access to finances for medicines and healthcare as well as access to transportation in many cases. It is possible that women may have had lower self-efficacy regarding healthcare seeking decisions because these decisions involved several variables outside the woman’s control and thus were associated with a more external locus of control.

Many of the decisions captured in our study are what some scholars would refer to as second-order or less significant decisions [6]. First-order decisions are strategic life choices and have far-reaching consequences (e.g., what job to take; when and how many children to have, etc.), but are relatively rare [6]. It could be argued, then, that the decision-making autonomy represented in our study is not as directly indicative of women’s empowerment as it might have been were the same measure to be applied to first-order decisions. It is possible that certain health and nutrition decisions, such as what foods to prepare for the family and whether to feed the children certain foods, may not be robust indications of women’s power, not only because of their nature as second-order decisions but because these decisions are often assigned to women in rural Bangladesh. However, autonomy in even these second-order decisions was significantly associated with self-efficacy. This finding is corroborated by other studies in similar contexts. In a qualitative examination of the process of empowerment for women participating in an HFP intervention in Nepal, self-efficacy emerged as the most consistent facilitating factor across all stages, even as nearly all women reported that they were able to decide which crops to grow, what techniques to apply, and which fertilizers to use [16]. This is a clear indication for researchers and practitioners interested in women’s decision-making agency that self-efficacy should not be overlooked and likely plays a critical role in facilitating women’s fulfillment of their preferences for participation in decision-making, even in choices that have not traditionally been considered to have a significant bearing on women’s power.

### Generalized vs. domain-specific self-efficacy

Among joint decision-makers, domain-specific self-efficacy was more strongly associated with decision-making autonomy than generalized self-efficacy. The degree of confidence women had in making specific decisions was more important than their general sense of self-efficacy in influencing the probability that women participated in those decisions according to their preferences. Some authors have argued that GSE is most relevant or dominant when participants are dealing with an unknown task or domain [20]. In these cases, the participant has not established other sources of information (mastery experiences, vicarious experiences, etc.) related that task and, instead, defaults to their generalized sense of self-efficacy [20]. As noted above, health and nutrition decisions were commonly assigned to women in our study context; therefore, SSE beliefs around these decisions would have likely been well-established leaving a smaller role for GSE to play. Additionally, the concept of specificity matching from the self-efficacy literature states that the measure of self-efficacy (either GSE or SSE) should be aligned with the degree of specificity of the outcome in question [18]. It follows, then, that SSE would be a better predictor of an outcome defined as narrowly as autonomy in decision-making related to one’s personal diet during pregnancy, for example. While the cross-sectional nature of our study does not allow for a robust understanding of the temporality or directionality between GSE and SSE, further research could examine whether GSE is perhaps an antecedent of SSE and/or whether a constellation of mastery experiences in various task domains may contribute to increased GSE. There is some evidence in the qualitative results presented above that self-efficacy in one task domain (e.g., gardening, livestock rearing) may be transferable to other, similar task domains.

### Strengths and limitations

This study had several key strengths including the validity and reliability of the measurement tools used for quantitative data collection. The New General Self-Efficacy scale has been empirically tested and demonstrated strong content validity and discriminant validity and was shown to be highly reliable and unidimensional [18]. The measure of SSE, by contrast, relied on one item per activity. We were limited to the available data from the health and nutrition module, however, future research should explore validation of SSE measures for health and nutrition decision-making (see [51] for an example of an occupational SSE scale). Tests of the pro-WEAI’s measurement properties found that items loaded as expected and fit indices were sufficient for the self-efficacy scale [9]. The health and nutrition module was cognitively validated in Bangladesh [52] and its dimensionality was assessed using data from six projects across Bangladesh, Burkina Faso, and Mali; results show that the factor model meets standards of acceptable fit across the projects [37]. The use of qualitative data in our study also lent greater validity to the interpretations of the quantitative findings. While many studies have assessed the influence of either or both intrinsic and instrumental agency on some outcome of interest [8, 53] the current study is one of the first to examine the associations between measures of intrinsic and instrumental agency [27]. However, we are unable to draw causal conclusions due to the cross-sectional nature of the study design.

The current study also had a number of important limitations. For analyses examining GSE as compared to SSE, we had to restrict our sample to only those women who reported making decisions jointly with other household members, as only joint decision-makers were asked about their confidence in making the given decision. This created sample size limitations for this portion of the analysis, particularly for the items related to pregnancy, which were further restricted to women who had been pregnant or given birth within the past two years. The analyses that were restricted to joint decision-makers may also have limited external validity in that the results can only speak to the influence of SSE on decision-making autonomy for those women who were participating in decision-making specifically as a joint activity with other family members. The experiences of those who make decisions alone are likely different from those who make decisions with others. Qualitative findings suggested that the involvement of additional household members (e.g., in-laws) in decision-making tended to increase the formality of the process and introduced additional restrictions to women’s agency; therefore, self-efficacy likely plays a more important role in joint (rather than sole) decisions. However, future researchers may choose to collect SSE information for both joint and sole decision-makers to enable quantitative comparisons. Additionally, women who were not at all involved in health and nutrition decisions (i.e., listed only other household members and not themselves when asked who usually makes the given decision) were excluded from our analysis. This sub-sample of women was too small to draw meaningful conclusions from but may have offered important insights with a larger sample size.

## Conclusion

The current study offers a solution to the challenges of empirically measuring women’s decision-making agency in a manner that directly recognizes and captures the role of women’s preferences regarding participation in decision-making. The study findings also have important implications for intervention design and evaluation. Development practitioners seeking to impact women’s agency should consider the role of self-efficacy in the empowerment process. We recommend further measurement of generalized and domain-specific self-efficacy at critical points throughout the theory of change in empowerment programming in order to better develop the evidence base around these aspects of intrinsic and instrumental agency. Development of longitudinal evidence can help build an understanding of how GSE, SSE, and decision-making agency may build upon or contribute to one another in various contexts.

### Supplementary Information

**Additional file 1:** **Supplementary Table A.** Domain-specific self-efficacy items.

## Data Availability

The quantitative data used and/or analyzed during the current study are available from the corresponding author on reasonable request.

## Abbreviations

Pro-WEAI: Project-level Women’s Empowerment in Agriculture Index
GSE: Generalized self-efficacy
SSE: Domain-specific self-efficacy
FAARM: Food and Agricultural Approaches to Reducing Malnutrition
GAAP2: Gender, Agriculture, and Assets Project Phase 2
HFP: Homestead food production
NGSE: New General Self-Efficacy [scale]
IDI: in-depth interview
SD: Standard deviation
OR: Odds ratio
CI: Confidence interval
P: Participant
I: Interviewer
