# Supplementary material for: The role of self-efficacy in women’s autonomy for health and nutrition decision-making in rural Bangladesh
Source: BMC Public Health. 2024 Feb 1;24:338. doi: 10.1186/s12889-024-17663-2 (PMC10832193; doi:10.1186/s12889-024-17663-2)
Supplement: Supplementary file 1 — Additional file 1: Supplementary Table A. Domain-specific self-efficacy items. [file 12889_2024_17663_MOESM1_ESM.docx]

| **Supplementary Table A**. Domain-specific self-efficacy items  *asked to women who were pregnant or had given birth within the past 2 years; †asked to women who had children at least 6 months of age; ‡ asked to women with children of any age | | |
| --- | --- | --- |
| **Decision-making category/index** | **Activity** | **Item**  *How confident do you feel to make decisions about…* |
| Personal health and diet | Rest when ill | *…how much you can rest when you are ill?* |
|  | Foods to prepare | *…what foods to prepare every day?* |
|  | Foods to eat | *…what foods (available in the house) you can eat?* |
| Personal diet during pregnancy | Eat eggs during pregnancy | *…whether you could eat eggs during your current or most recent pregnancy?** |
|  | Consume milk during pregnancy | *…whether you could consume milk or milk products during your current or most recent pregnancy?** |
|  | Eat meat during pregnancy | *…whether you could eat meat, poultry or fish during your current or most recent pregnancy?** |
| Personal health during pregnancy | Work during pregnancy | *…how much you worked during your current or most recent pregnancy?** |
|  | Rest during pregnancy | *…how much you could rest during your current or most recent pregnancy?** |
|  | Consult a doctor during pregnancy | *…whether you consulted a doctor or went to a clinic during your current or most recent pregnancy?** |
| Child’s diet | Feed child eggs | *…whether or not your child is offered eggs to eat?†* |
|  | Feed child milk | *…whether or not your child is offered milk or milk products, other than breastmilk?†* |
|  | Feed child meat | *…whether or not your child is offered meat, poultry or fish?†* |
| Healthcare seeking | Go to the doctor when ill | *…whether or not you consult a doctor or go to a clinic when you are ill?* |
|  | Take sick child to doctor | *…whether your child is taken to a clinic or a doctor is consulted when he/she is sick? ‡* |
|  | Take child for well visits | *…whether your visits the health clinic to see if he/she is growing well? ‡* |
